# Immunoglobulin G glycome composition in transition from premenopause to postmenopause

**DOI:** 10.1016/j.isci.2022.103897

**Published:** 2022-02-10

**Authors:** Helena Deriš, Domagoj Kifer, Ana Cindrić, Tea Petrović, Ana Cvetko, Irena Trbojević-Akmačić, Ivana Kolčić, Ozren Polašek, Louise Newson, Tim Spector, Cristina Menni, Gordan Lauc

**Affiliations:** 1 Genos Glycoscience Research Laboratory, Zagreb 10000, Croatia; 2 Faculty of Pharmacy and Biochemistry, University of Zagreb, Zagreb 10000, Croatia; 3 University of Split School of Medicine, Split 21000, Croatia; 4 Algebra University College, Zagreb 10000, Croatia; 5 Newson Health Menopause & Wellbeing Centre, Church Street, Stratford-Upon-Avon CV37 6HB, UK; 6 Department of Twin Research and Genetic Epidemiology, King’s College London, Westminster Bridge Road, SE17EH London, UK

**Keywords:** Reproductive medicine, Molecular biology, Glycomics

## Abstract

Gonadal hormones affect immunoglobulin G (IgG) glycosylation, and the more proinflammatory IgG glycome composition might be one of the molecular mechanisms behind the increased proinflammatory phenotype in perimenopause. Using ultra-high-performance liquid chromatography, we analyzed IgG glycome composition in 5,080 samples from 1940 pre-, peri-, and postmenopausal women. Statistically significant decrease in galactosylation and sialylation was observed in postmenopausal women. Furthermore, during the transition from pre- to postmenopausal period, the rate of increase in agalactosylated structures (0.051/yr; 95%CI = 0.043–0.059, p < 0.001) and decrease in digalactosylated (−0.043/yr; 95%CI = −0.050 to −0.037, p < 0.001) and monosialylated glycans (−0.029/yr; 95%CI = −0.034 to −0.024, p < 0.001) were significantly higher than in either pre- or postmenopausal periods. The conversion to the more proinflammatory IgG glycome and the resulting decrease in the ability of IgG to suppress low-grade chronic inflammation may be an important molecular mechanism mediating the increased health risk in perimenopause and postmenopause.

## Highlights

• Different levels of IgG N-glycans in premenopausal and postmenopausal women and men
• Perimenopause causes a higher rate of increase in agalactosylated glycan structures
• Perimenopause causes faster decrease of digalactosylated and monosialylated glycans
• Proinflammatory IgG glycome may mediate the increased health risk in perimenopause

## Graphical abstract

**Figure:**
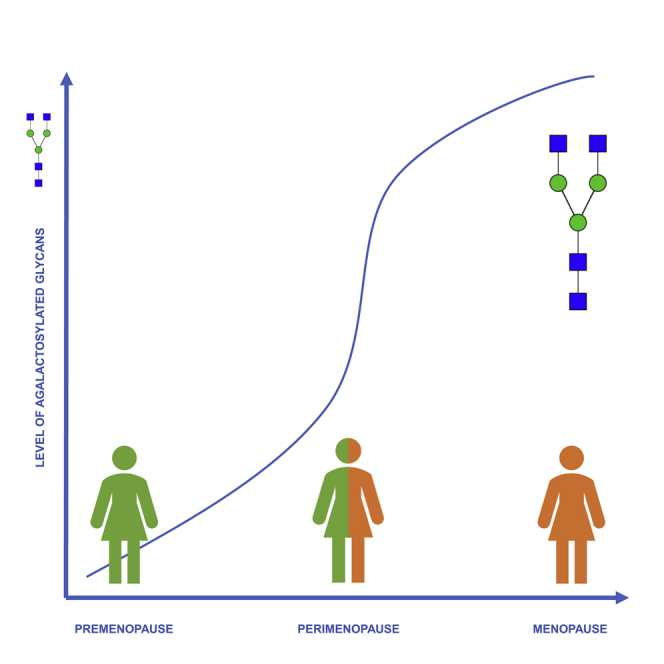

## Introduction

Glycosylation of immunoglobulin G (IgG) is an important regulator of the immune system (Martinić Kavur et al., 2021). In addition to directly affecting the effector functions of IgG by promoting its binding to different Fc receptors (Nimmerjahn and Ravetch, 2008a), glycans attached to IgG have numerous other roles in the regulation of the immune system (Ipsen-Escobedo and Nimmerjahn, 2018; Seeling et al., 2017). Glycans are also important mediators of the anti-inflammatory activity of IgG (Karsten et al., 2012; Schwab and Nimmerjahn, 2013) as well as the driving force for one of the molecular mechanisms underlying immunosuppressive activity of intravenous IgG (IVIG) therapy used to treat a variety of immunological disorders (Nimmerjahn and Ravetch, 2008b). Changes in IgG glycosylation are also believed to be an important contributor to aging at the molecular level through a process called inflammaging (Franceschi et al., 2018). Furthermore, hyposialylated IgG, which associates with ageing, was shown to contribute to the development of hypertension in animal models through FcγRII on endothelial cells (Peng et al., 2019; Sundgren et al., 2015).

IgG glycome composition is tightly regulated and, despite the absence of a direct genetic template, is heritable to a significant extent (Menni et al., 2013; Pučić et al., 2011). Alternative glycosylation (the attachment of different glycan structures to the same glycosylation site) is structurally and functionally analogous to coding mutations, but instead of being defined by sequence variation in individual genes, glycome composition is inherited as a complex trait encoded by multiple genes (Krištić et al., 2018). Genome-wide association studies identified a network of over 30 genes that associate with the IgG glycome composition (Klarić et al., 2020; Lauc et al., 2013; Shen et al., 2017). One of the regulators of IgG glycosylation are sex hormones, and the association between estradiol and the IgG glycome composition was confirmed to be causal (Ercan et al., 2017). A recent randomized placebo-controlled clinical study demonstrated that the deprivation of gonadal hormones resulted in an increase of biological age measured by IgG glycans, which was completely prevented by estradiol supplementation (Juric et al., 2020).

Large population studies revealed that IgG glycome composition changes with age and that in females this change is particularly pronounced in the time preceding the average age of menopause (Krištić et al., 2014; Stambuk et al., 2020). However, the association between changes in the IgG glycome composition and menopause has not yet been validated. Perimenopause is accompanied by different symptoms and is very often misdiagnosed due to changing hormone levels and symptom overlap with other conditions. Menopause associates with an increased risk of developing a range of diseases affecting metabolism, bones, cardiovascular system, brain, and even cancer (Lobo et al., 2014). Early perimenopause diagnosis would enable timely interventions to ease the symptoms, decrease risks of developing accompanying diseases, and timely monitoring of any changes in women's health status due to hormonal changes. To fill this knowledge gap and explore the association of IgG glycome changes with perimenopause, we analyzed IgG N-glycome composition in 5,080 samples from 1,940 females in multiple time points during their transition from premenopause to postmenopause, along with 274 samples from 113 males. As an additional control, samples from 301 females in an independent validation cohort were analyzed.

## Results

### IgG glycome composition in premenopause and menopause

**Figure 1:**
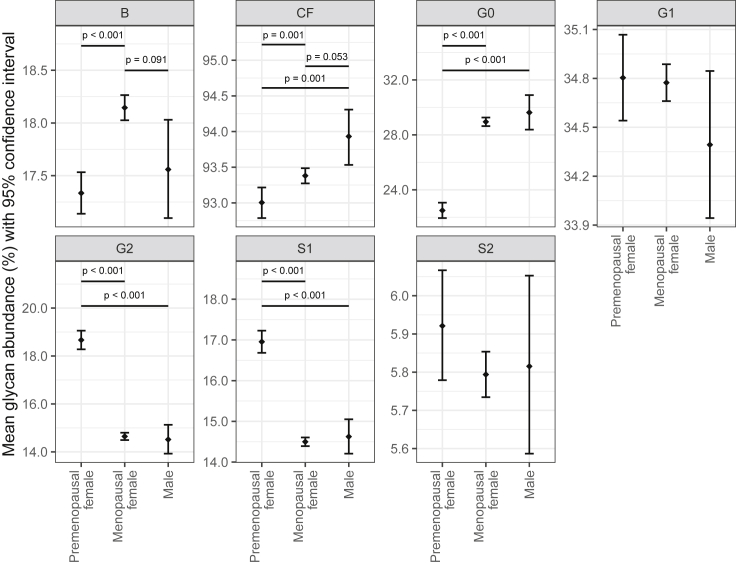
IgG glycome composition in premenopausal women, menopausal women, and men Mean glycan abundances (% of total IgG glycome) and corresponding 95% confidence intervals were estimated from the fitted mixed model with logit transformed glycan as dependent variable and sex, menopausal status (nested within sex), age, and age-menopausal status interaction as fixed factors, as well as family ID and individual ID (nested within family ID) as random intercepts and age as random slope. Only adjusted post-hoc p values less than 0.1 are shown. B, bisecting N-acetylglucosamine (GlcNAc); CF, core fucose; G0, agalactosylated glycans; G1, monogalactosylated glycans; G2, digalactosylated glycans; S1, monosialylated glycans; and S2, disialylated glycans.

IgG glycome composition was analyzed in multiple samples from 1,940 females and 113 males from the UK registry of adult twins. The 1,087 samples from 732 individual twins (500 families) were collected prior to the onset of menopause, whereas 3,993 samples from 1,678 individual twins (969 families) were collected after the onset of menopause (Table S1). In addition, IgG glycome was analyzed in 113 men (274 samples) as an additional control. A representative chromatogram with detailed structures of individual IgG glycans is shown in Figure S1. Mixed modelling was used to determine average levels of individual IgG glycans in premenopausal women, menopausal women, and men. Large and statistically significant differences were observed in multiple glycans, mainly reflecting decreased galactosylation and sialylation of glycans in menopausal women (Figure 1). Levels of individual IgG glycans are available in Figure S2.

### Average yearly change of IgG glycans in perimenopause

**Figure 2:**
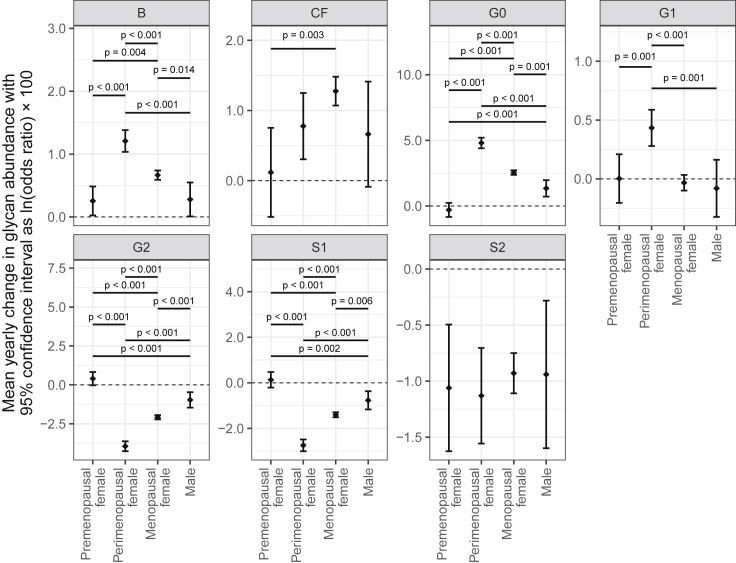
Average yearly change of IgG glycans in females during the perimenopause period, females in pre- or postmenopause, and males Mean yearly change in IgG glycan abundances and corresponding 95% confidence intervals were estimated from the fitted mixed model with logit transformed glycan as dependent variable and sex, menopausal status (nested within sex), age and age-menopausal status interaction as fixed factors, as well as family ID and individual ID (nested within family ID) as random intercepts and age as random slope. Only adjusted post-hoc p values less than 0.1 are shown. B, bisecting GlcNAc; CF, core fucose; G0, agalactosylated glycans; G1, monogalactosylated glycans; G2, digalactosylated glycans; S1, monosialylated glycans; and S2, disialylated glycans.

It is known that IgG galactosylation and sialylation decrease with age (Krištić et al., 2014), thus we attempted to evaluate whether the changes in glycans associated with the transition to menopause were more extensive than the age-related changes. Because we had multiple samples from the same individual, we were able to calculate the rate of change of individual IgG glycans over time. A subset of women in our cohort (n = 379) entered menopause between two sampling time points, thus for them, we were also able to calculate the rate of changes in individual IgG glycans during the perimenopausal period. The comparison of the rate of age-related IgG glycome changes (when there was no change of the menopause status) with the rate of IgG glycome changes during perimenopause (i.e., between time points when the transition to menopause occurred) revealed statistically significant differences for a number of glycans (Figure S3). The most prominent differences in the perimenopause period were the significantly higher rates of increase in the agalactosylated structures (G0) and the decrease of digalactosylated (G2) and monosialylated (S1) glycans (Figure 2). During perimenopause women had a significant higher rate of increase in agalactosylated structures (0.051/yr; 95%CI = 0.043–0.059, p < 0.001) and decrease in digalactosylated (−0.043/yr; 95%CI = −0.050 to −0.037, p < 0.001) and monosialylated glycans (−0.029/yr; 95%CI = −0.034 to −0.024, p < 0.001), compared with premenopausal women.

### Prediction of perimenopause using IgG glycans

**Figure 3:**
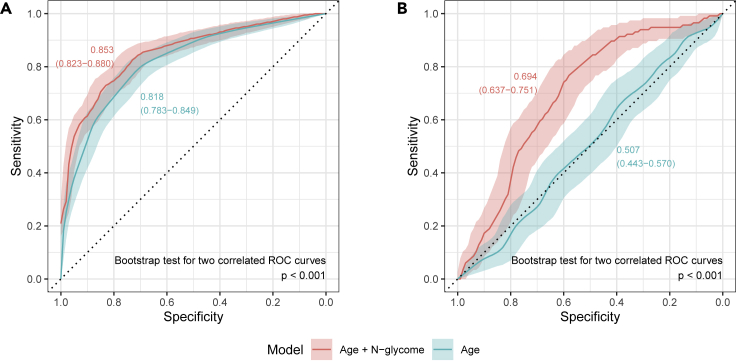
Receiver operating characteristic (ROC) curves for the prediction of perimenopause IgG glycans measured in a single time point (A) and the change in IgG glycan levels between the two time points (B). Graphs are showing the area under the curve (AUC) values with 95% confidence intervals.

Using single-point measurements of IgG N-glycans we attempted to predict menopause status. The lasso model was fitted with menopausal status (TRUE for those who entered menopause and FALSE for all others) as dependent variable and age and all directly measured IgG glycans as predictors. The model was fitted on a randomly chosen one twin per family (training set) in the subset of women with a two time point median age between 45 and 55 years. The remaining twins were used for testing the fitted model. The same train and test subset model with only mean age as a predictor were used to fit and test the model for comparison (Figure 3A). IgG N-glycome in combination with age measured in a single time point has shown a good classification performance, with the AUC of 0.853 (95% CI 0.823–0.880) outperforming only age as a predictor of perimenopause (AUC of 0.818, 95% CI 0.783–0.849). Because changes in the IgG glycome composition were the most prominent in the period of perimenopause, we used the rate of changes in glycans to predict whether given women already entered the period of perimenopause or not (Figure 3B). The addition of the information about changes in the IgG glycan levels to a prediction model notably increases AUC value compared with using only age (0.694, 95% CI 0.637–0.751 compared with 0.507, 95% CI 0.443–0.570, respectively).

### IgG glycome composition in replication cohorts

**Figure 4:**
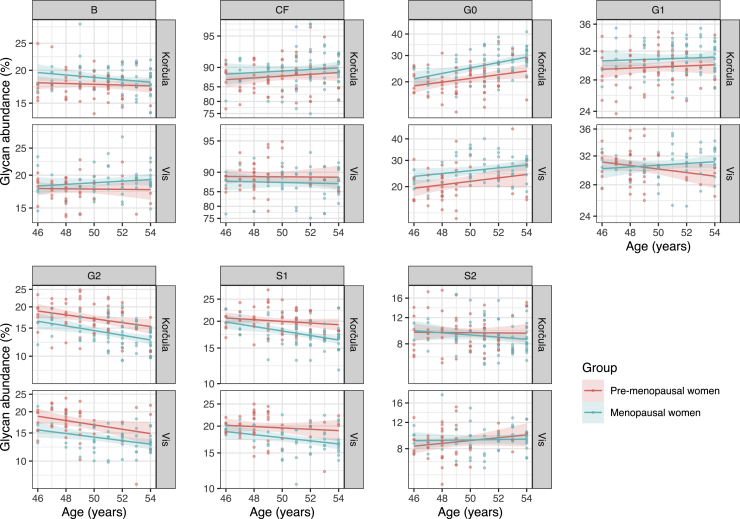
IgG glycome composition in premenopausal and menopausal women of any age between 45 and 55 years in replication cohorts Glycan abundance (%) is presented as % of total IgG glycome Lines are representing mean levels of glycan abundance by age for each cohort with SE of mean presented as the shaded area around the mean. B, bisecting GlcNAc; CF, core fucose; G0, agalactosylated glycans; G1, monogalactosylated glycans; G2, digalactosylated glycans; S1, monosialylated glycans; and S2, disialylated glycans.

The sampling in replication cohorts was cross-sectional, and there was only one time point for each sample, which did not allow to infer any causal relationships such as monitoring changes in IgG N-glycome over time. Because of that limitation, this was not a perfect replication cohort for our model validation; therefore, it was not possible to assess whether someone is perimenopausal or not. What we could replicate are the consistent differences between premenopausal and menopausal females of any age between 45 and 55 years (Figure 4).

## Discussion

By analyzing IgG glycome in multiple samples from the same individuals, we have shown the association between the period of perimenopause and the extensive changes in the IgG glycome composition. Aging associates with the transition of the IgG glycome from an inflammation-suppressive to a proinflammatory composition, but this change is much faster in the period when women are progressing from a regular cycle to menopause, as was shown in several population studies (Krištić et al., 2014; Stambuk et al., 2020). The most extensive change in the IgG glycome during perimenopause was a decrease in galactosylation and sialylation, a change that is generally associated with aging (Martinić Kavur et al., 2021). The depletion of estrogen during perimenopause and menopause is an important component of aging, as low hormone levels increase vulnerability to diseases in hormone-responsive tissues, including the brain, bone, and the cardiovascular system (Lobo et al., 2014). Menopause is, therefore, more complex than just being a natural condition resulting in symptoms; it needs to be considered as a long-term hormone deficiency potentially leading to higher health risks if not appropriately managed with lifestyle adjustments or hormone replacement therapy (HRT) in consonance with a gynecologist and/or general practitioner (Gambacciani et al., 2019).

The age-related gradual decrease in the level of galactosylated IgG and the increase in agalactosylated IgG is known to exacerbate low-grade chronic inflammation (De Martinis et al., 2005; Franceschi et al., 2000; Monti et al., 2017) and acts as an effector of proinflammatory pathological changes (Dall’Olio et al., 2013; Franceschi et al., 2007). Decreased levels of IgG galactosylation and/or sialylation have been associated with a wide range of autoimmune conditions, such as systemic lupus erythematosus, inflammatory bowel disease, and many others. Changes in these IgG glycosylation traits are also associated with disease progression, disease activity, symptom severity, and response to treatment. In many cases, these changes occur before the onset of symptoms (Gudelj et al., 2018a).

The rate of change from “young” to “old” IgG glycome increased significantly in the perimenopausal period, suggesting that the loss of immunosuppressive functions of IgG may be an underlying molecular mediator of at least some health risks in this period. The results of this study also indicate the potential of the IgG glycome composition as a biomarker for perimenopause. Perimenopause can last up to 15 years and is difficult to diagnose due to highly irregular hormonal cycles (National Institute for Health and Care Excellence, 2015). As a result of poor awareness and inappropriate use of hormonal tests, women are often misdiagnosed with conditions such as fibromyalgia, migraines, depression, or chronic fatigue syndrome and are frequently prescribed antidepressants despite there being no evidence to support their use to improve the low mood associated with perimenopause or menopause (Leonhardt, 2019). The importance of early diagnosis of perimenopause is supported by the research that has shown that early use of HRT leads to a greater reduction of future disease incidence as well as minimizing the duration of symptoms (Ward and Deneris, 2018).

Recent placebo-controlled randomized trials demonstrated the importance of estrogens in the regulation of IgG glycosylation (Ercan et al., 2017; Juric et al., 2020), thus it is reasonable to assume that the decrease in estrogen levels is an important driver of changes in IgG glycosylation in perimenopause. Contrary to estrogens that have a very short half-life in circulation, IgG has a half-life of 3 weeks (Vidarsson et al., 2014), which implies that the current composition of the IgG glycome is affected by average concentrations of estrogen in the past 3–4 weeks. Because changes in the IgG glycome during perimenopause are so extensive, they could potentially be used as a diagnostic tool. In fact, IgG glycome composition could potentially be used to determine long-term average concentrations of sex hormones in the same way glycated hemoglobin (HbA1c) is used to determine long-term concentrations of blood glucose.

## Conclusions

Changes in IgG glycosylation from “young” to “old” glycome associate with many health risks that accompany menopause (Martinić Kavur et al., 2021). In some diseases such as rheumatoid arthritis (Ercan et al., 2010; Gudelj et al., 2018b) and cardiovascular diseases (Kifer et al., 2021; Menni et al., 2018; Wittenbecher et al., 2020), this change was shown to occur years before disease onset, suggesting the causal role of IgG glycans in disease development. Proinflammatory IgG glycome and the resulting decrease in the ability of IgG to suppress low-grade chronic inflammation may be an important molecular mechanism that mediates the increased health risk in perimenopause, thus this topic should be studied in more detail.

### Limitations of the study

The main limitation of this study is the relatively long time between individual sampling time points (6.9 ± 2.6 years) that was not tailored for the occurrence of menopause. Therefore, in some cases, perimenopause was just a short part of the period in which the change in the IgG N-glycome occurred, whereas in others sampling might have occurred within the perimenopause period, with part of the perimenopause being before and another part after a specific sampling. Hopefully, a future study that would have more frequent sampling during the perimenopause period would enable an even more accurate assessment of IgG glycome association with perimenopause.

## STAR★Methods

### Key resources table

**Table:** 

REAGENT or RESOURCE	SOURCE	IDENTIFIER
**Biological samples**		

Human serum samples	TwinsUK Registry	http://www.twinsuk.ac.uk
Human plasma samples, CROATIA-Korčula	“10001 Dalmatians” – Croatian National Biobank (Krištić et al., 2014)	http://www.mefst.unist.hr/znanost/istrazivacke-skupine-i-laboratoriji/10-001-dalmatinac-hrvatska-biobanka/5035
Human plasma samples, CROATIA-Vis	“10001 Dalmatians” – Croatian National Biobank (Krištić et al., 2014)	http://www.mefst.unist.hr/znanost/istrazivacke-skupine-i-laboratoriji/10-001-dalmatinac-hrvatska-biobanka/5035

**Chemicals, peptides, and recombinant proteins**		

Protein G monolithic 96-well plate	BIA Separations	Pucic et al. 2011
Acetonitrile, LC/MS grade	Honeywell	CAS#75-05-8, EC#200-835-2
Formic acid	Merck	Cat#100264, CAS#64-18-6, EC#200-579-1
Ammonium bicarbonate	Sigma Aldrich	Cat#09830, CAS#1066-33-7, EC#213-911-5
2-aminobenzamide (2-AB)	Sigma Aldrich	CAS# 88-68-6, EC# 201-851-2
Dimethyl sulfoxide (DMSO)	Sigma Aldrich	CAS# 67-68-5, EC# 200-664-3
Glacial acetic acid	Merck	CAS# 64-19-7, EC#200-580-7
1xPBS (phosphate buffer saline); prepared from:	prepared in-house	
NaCI (Sodium chloride)	Alkaloid Skopje	CAS#7647-14-5, EC#231-598-3
Na_2_HPO_4_ (Disodium phosphate)	BIOCHEM Chemopharma	CAS#7558-79-4, EC#231-448-7
KH₂PO₄ (Monopotassium phosphate)	BIOCHEM Chemopharma	CAS#7778-77-0, EC#231-913-4
KCl (Potassium chloride)	Sigma Aldrich	CAS#7447-40-7, EC#231-211-8

**Critical commercial assays**		

GlycoWorks RapiFluor-MS N-Glycan Kit	Waters Corporation	Cat#176003910

**Software and algorithms**		

Empower 3 software, build 3471	Waters Corporation	https://www.waters.com/waters/promotionDetail.htm?id=10184996&locale=en_US
R programming language	R Core Team	https://www.r-project.org/
R package ‘sva’	(Leek et al., 2012)	https://bioconductor.org/packages/release/bioc/html/sva.html
R package ‘lme4’	(Bates et al., 2015)	https://cran.r-project.org/web/packages/lme4/index.html
R package ‘emmeans’	(Lenth, 2020)	https://cran.r-project.org/web/packages/emmeans/index.html
R package ‘caret’	(Kuhn, 2020)	https://cran.r-project.org/web/packages/caret/vignettes/caret.html
R package ‘glmnet’	(Friedman et al., 2010)	https://cran.r-project.org/web/packages/glmnet/index.html
R package ‘pROC’	(Robin et al., 2011)	https://cran.r-project.org/web/packages/pROC/index.html

### Resource availability

#### Lead contact

The lead contact for further information and requests is Gordan Lauc (glauc@pharma.hr).

#### Materials availability

The study did not generate new unique reagents.

### Experimental model and subject details

#### Subject details

Study subjects were individuals enrolled in the TwinsUK registry, a national register of adult twins recruited as volunteers without selecting for any particular disease or traits (Verdi et al., 2019). Data on menopause was gathered through the multiple-choice questionnaire asking women at each visit: “What is your menopausal status?” with possible answers “Premenopausal”, “Going through the menopause”, “Postmenopausal” and “Don’t know” and “How old were you when you became postmenopausal (when you stopped having periods for one year or more)?”. Consequently, a sample was defined as menopausal if the questionnaire resulted in a “Postmenopausal” answer or age greater than the reported age of becoming postmenopausal. Similarly, a sample was defined as premenopausal if the questionnaire resulted with a “Premenopausal” answer or age at sampling was less than the reported age of becoming postmenopausal reduced by 1 year. Any inconsistent (i.e. “Premenopausal” answer at age after becoming menopausal) or unreliable data (i.e. answered as “Going through the menopause”) were dropped from the study (Table S1). The TwinsUK study was approved by NRES Committee London–Westminster, and all twins provided informed written consent.

#### Validation cohort

The validation cohort consisted of two different population studies, CROATIA-Vis and CROATIA-Korcula, described elsewhere in detail (Polašek et al., 2009; Rudan et al., 2009; Vitart et al., 2008). These studies were part of a larger “10 001 Dalmatians” study of Croatian island isolates, in this case, islands of Vis and Korcula, within the scope of the Croatian National Biobank. All human participants included in this study signed informed consent and the study was approved by the appropriate Ethics Committee of the University of Split Medical School.

### Method details

#### Isolation of IgG

IgG isolation from serum samples was performed on protein G monolithic 96-well plates (Trbojević-Akmačić et al., 2017), 100 μL of serum was diluted 1:7 (v/v) with 1xPBS (phosphate buffer saline) followed by filtration on a 0.45 μm GHP filter plate (Pall Corporation, USA). Diluted and filtered serum samples were then transferred to the protein G monolithic plate (BIA Separations, Slovenia), which was then washed three times with 2 ml 1xPBS. Elution of IgG was performed with 1 mL of 0.1 mol/L formic acid (Merck, Germany) followed by immediate neutralization of the mixture with ammonium bicarbonate (Acros Organics, USA) to pH 7.0. IgG-containing eluate was aliquoted into a PCR plate (average mass of 15 μg of IgG per sample) and dried in a vacuum centrifuge.

#### Deglycosylation, labelling and purification of IgG N-glycans

Deglycosylation, RapiFluor-MS labelling and purification of IgG N-glycans were performed using the GlycoWorks RapiFluor-MS N-Glycan Kit obtained from Waters Corporation (USA). The entire procedure was done following Waters’ protocol (Waters Corporation, 2017, https://www.waters.com/webassets/cms/support/docs/715004793en.pdf), which was further adapted to suit the high-throughput analysis in the 96-well PCR plate format (Deriš et al., 2021). Isolated and dried IgG was first resuspended in 10.8 μL ultrapure water, swiftly denatured with 3 μL 5 % RapiGest SF solution in a 3 min reaction at 99°C and enzymatically deglycosylated with 1.2 μL GlycoWorks Rapid PNGase F in a 5 min reaction at 50°C. Released N-glycans were then labeled with the RapiFluor-MS label. RapiFluor-MS dye contains a rapid tagging function group, N-hydroxysuccinimide carbamate, which rapidly modifies glycosylamine-bearing N-glycans after their enzymatic release, yielding a stable urea linkage (Lauber et al., 2015). Labeled N-glycans were purified by hydrophilic interaction chromatography-solid phase extraction (HILIC-SPE) clean-up. In the end, the samples of released and labelled IgG glycans were stored at -20 °C until further use.

#### HILIC-UHPLC-FLR analysis of RapiFlour-MS labelled IgG N-glycans

RapiFluor-MS labeled IgG N-glycans were analysed using ultra-high-performance liquid chromatography based on hydrophilic interactions with fluorescence detection (HILIC-UHPLC-FLR) on Waters Acquity UPLC H-class instruments. The instruments were controlled and monitored with the Empower 3 software, build 3471 (Waters, USA). Chromatographic separation of glycan structures was performed on Waters UPLC Glycan bridged ethylene hybrid (BEH) Amide chromatographic columns (130 Å, 1.7 μm BEH particles, 2.1 × 100 mm). Solvent A was 50 mmol/L ammonium formate, pH 4.4, while solvent B was 100% LC-MS grade acetonitrile. A linear gradient of 75–61.5 % acetonitrile (v/v) was used at a flow rate of 0.4 mL/min over 30 min in a 42-min analytical run. Each chromatogram was separated into 22 glycan peaks containing IgG glycan structures (Figure S1.), by automated integration (Agakova et al., 2017) resulting in relative quantification of IgG N-glycans (total area normalization). Specific N-glycan structures found in each glycan peak according to Keser et al. (2018) are presented in Table S2.

#### Validation cohort

IgG was immobilized in a block of sodium dodecyl sulfate–polyacrylamide gel and N-glycans were released by digestion with PNGase F (ProZyme, Hayward, CA), in a 96-well microtiter plate to achieve the best throughput. Released N-glycans were then labeled with 25 μL fluorescent 2-aminobenzamide (2-AB) dye dissolved in dimethyl sulfoxide (DMSO) and glacial acetic acid mixture (85:15, v/v). The excess label was removed by solid-phase extraction using Whatman 3MM chromatography paper (Royle et al., 2008). Labeled glycans were eluted with water and stored at −20°C until further use. UHPLC analysis was performed as described previously (Krištić et al., 2014); 2-AB labeled IgG N-glycans were separated on a Waters UPLC Glycan BEH Amide chromatographic columns with 100 mmol/L ammonium formate, pH 4.4, as solvent A and 100% LC-MS grade acetonitrile as solvent B. Separation method used a linear gradient of 75–62% acetonitrile (v/v) at a flow rate of 0.4mL/min in a 25-min analytical run. Chromatograms were separated into 24 glycan peaks.

### Quantification and statistical analysis

#### Statistical analysis

After UHPLC analysis, each measured glycan peak was normalized by total chromatogram area. Observed relative glycan abundances were log transformed and batch corrected using ComBat method (R package ‘sva’ (Leek et al., 2012)). After batch correction, values were back-transformed to relative abundance and derived traits were calculated according to formulas presented in Table S3.

Overlapping measured N-glycomes and information available on menopause found in TwinsUK register resulted in 5354 samples from 1940 females and 113 males.

For each glycan trait, the effect of menopause on the abundance was estimated by mixed modelling (R package ‘lme4’ (Bates et al., 2015)). Logit transformed relative abundance was used as dependent variable and sex, menopausal status (nested within sex), age and age-menopausal status interaction as fixed factors, as well as family ID and individual ID (nested within family ID) as random intercepts and age as random slope. Using the model, mean values were estimated for each group and pairwise compared using t-test (post-hoc) (R package ‘emmeans’ (Lenth, 2020)). Given multiple testing for a large number of glycans, the control of false discovery rate (FDR) was done by adjusting p values according to Benjamini-Hochberg’s method.

For the analysis of the rate of change of glycan abundances, average yearly changes were calculated from two time points according to the formula: rate = (logit(glycan_2_) – logit(glycan_1_))/(age_2_ – age_1_), where glycan is relative abundance resulted from normalized chromatogram, and age is represented in years, assuming age_2_ > age_1_. Depending on menopausal status in the first and second time point measurements were classified into three categories: premenopausal (both timepoints before menopause), perimenopausal (first timepoint before, and second in the menopause), and menopausal (both timepoints in menopause). The diagram clarifying which time points were used to calculate the rate of change in IgG-N-glycome composition is shown in Figure S4. Mean rates were estimated by the mixed model, in which rate was dependent variable, and sex, menopausal group (nested within menopausal status) and mean age (calculated as (age_2_+age_1_)/2) were set as fixed factors, as well as family ID and individual ID (nested within family ID) as random intercepts. Estimated mean rates for each menopausal group and males were pairwise compared using a t-test (post-hoc). FDR control was done by adjusting p values according to Benjamini-Hochberg’s method.

N-glycome (observed in one timepoint) potential for diagnosis of perimenopause was analysed in a subset of women aged between 45 and 55 years by logistic regression with the L1 regularization technique (R package ‘caret’ and ‘glmnet’ (Friedman et al., 2010; Kuhn, 2020)). The subset was split into training (randomly chosen one measurement per family) and test (everything else) subset. The logistic model was defined with menopausal status (TRUE/FALSE) as the dependent variable, and age and all directly measured glycans as independent variables. To avoid overfitting, coefficients of the logistic model were estimated by 10-fold cross validation on a training subset data, with hyperparameter lambda set to lambda_min_. The model was tested on a test subset; results are presented by receiver operating characteristic (ROC) curve. In parallel, the null model was fitted in the same way but using only age as a predictor. Observed ROC curves of the two models were compared by a bootstrap test (B = 2000) (R package ‘pROC’ (Robin et al., 2011)).

The rate of N-glycome change (based on two time points) potential for diagnosis of perimenopause was analysed in a subset of women with median two-timepoint age between 45 and 55 years in the same way as diagnosis of menopause with IgG glycome analysed in one timepoint. The perimenopausal category was defined as TRUE, while others (postmenopausal and premenopausal) were defined as FALSE. All statistical analyses were done using R (R Core Team, 2020).

#### Validation cohort

For the following statistical analysis, only female samples of the matching ages, 45 to 55 years of age, were selected, which included 62 premenopausal females in the Vis study and 86 in the Korcula study and 70 and 83 menopausal females in the Vis and Korcula study respectively, as presented in the Table S4. Menopausal status was projected from a survey in which women indicated the age of becoming menopausal or answered the question (YES / NO) whether they had a regular menstrual cycle. Also, all women who use hormone replacement therapy (HRT) were excluded from the analysis. For each glycan trait, the effect of menopause on the abundance was estimated by mixed modelling (R package ‘lme4’ (Bates et al., 2015)). Logit transformed relative abundance was used as the dependent variable and menopausal status and age as fixed factors, as well as cohort ID as a random intercept. Using the model, mean values were estimated for each menopausal status group and compared using t-test (post-hoc) (R package ‘emmeans’ (Lenth, 2020)). Given multiple testing for the number of glycans, the control of false discovery rate (FDR) was done by adjusting p values according to Benjamini-Hochberg’s method.

## Data Availability

• The raw data reported in this study cannot be deposited in a public repository because the data are confidential records. To request access to data from the Twins UK Registry contact data access manager Victoria Vazquez (https://twinsuk.ac.uk/resources-for-researchers/access-our-data/) and to access the data from Vis and Korčula studies, contact Ozren Polašek (ozren.polasek@mefst.hr). • This paper does not report original code. • Any additional information required to reanalyze the data reported in this paper may be available from the lead contact upon request.
