# Supplementary material for: Immunoglobulin G glycome composition in transition from premenopause to postmenopause
Source: iScience. 2022 Feb 10;25(3):103897. doi: 10.1016/j.isci.2022.103897 (PMC8881712; doi:10.1016/j.isci.2022.103897)
Supplement: Document S1. Figures S1–S4 and Tables S1–S4 [file mmc1.pdf]

iScience, Volume *25*

Supplemental information

Immunoglobulin G glycome composition

in transition from premenopause to postmenopause

Helena Deriš, Domagoj Kifer, Ana Cindrić, Tea Petrović, Ana Cvetko, Irena Trbojević-Akmačić, Ivana Kolčić, Ozren Polašek, Louise Newson, Tim Spector, Cristina Menni, and Gordan Lauc

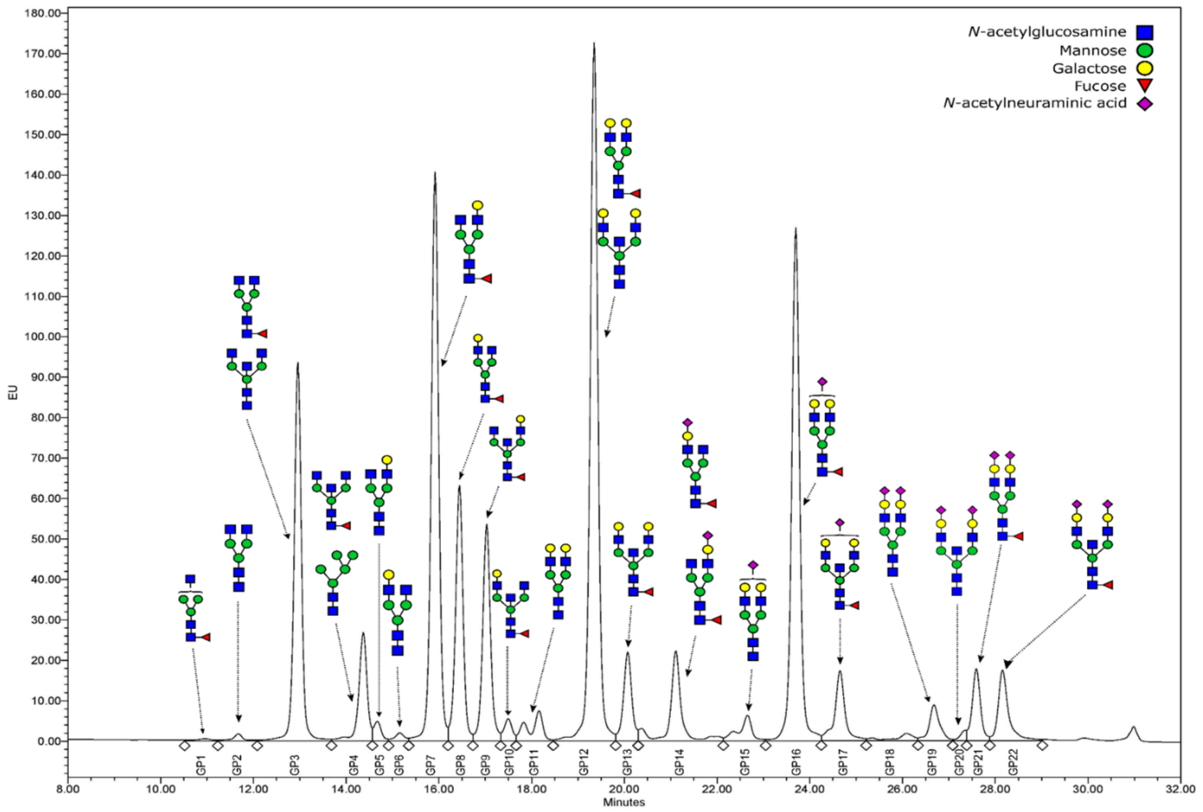

Figure S1. Representative HILIC-UPLC-FLR chromatographic profile of the immunoglobulin G RapiFluor-MS labeled N-glycome. Related to Figure 1 and Figure 2.

Graphic representation of the glycan structures corresponding to each glycan peak (GP). In the case of multiple structures per GP, the upper structure is the major one, and the lower one is minor in abundance. EU = emission units.

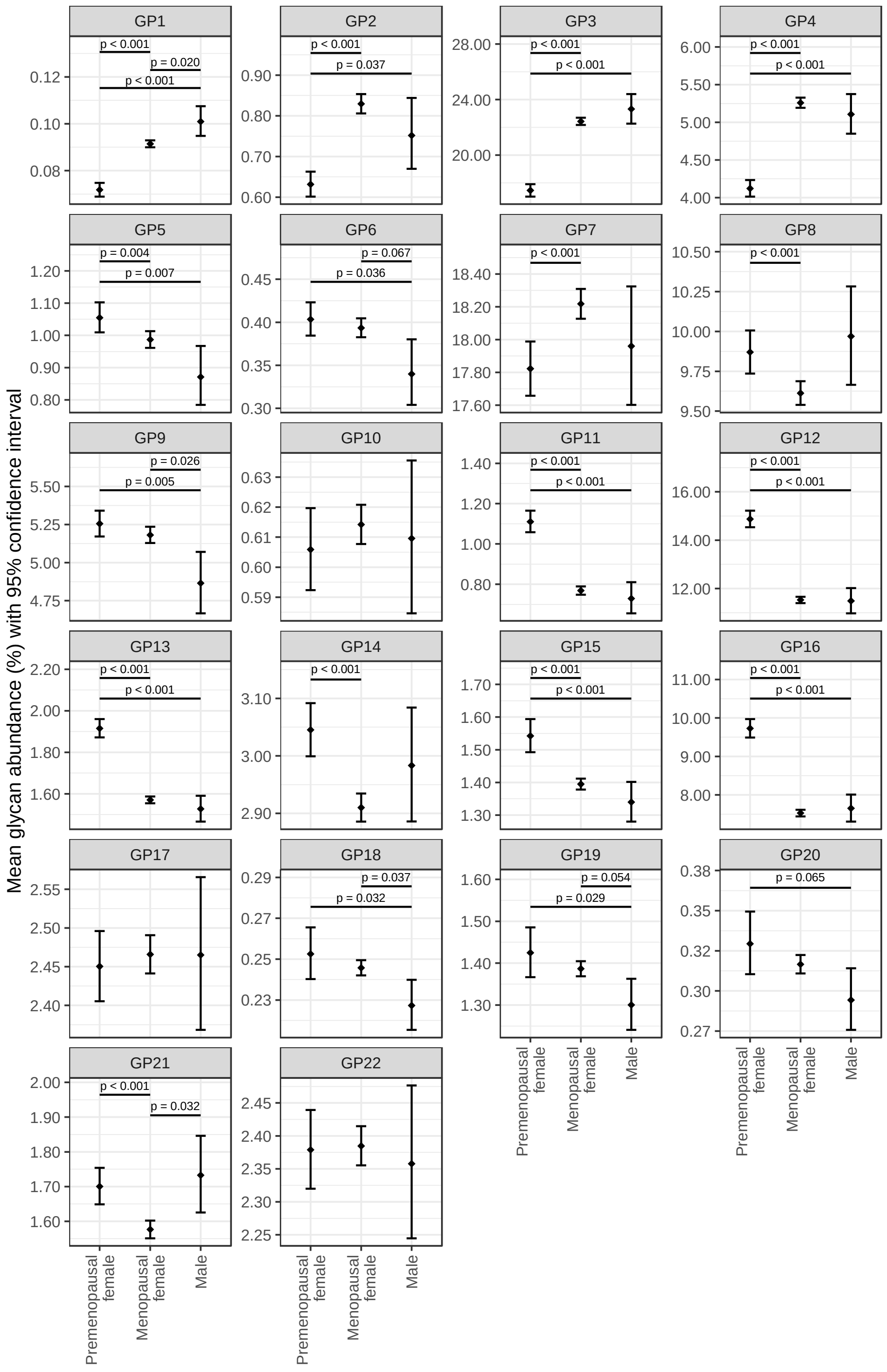

Figure S2. IgG glycome composition in premenopausal and postmenopausal women, and men. Related to Figure 1.

Mean glycan abundances (% of total IgG N-glycome) and corresponding 95% confidence intervals were estimated from the fitted mixed model with logit transformed glycanas dependent variable and sex, menopausal status (nested within sex), age and age-menopausal status interaction as fixed factors, as well as family ID and individual ID (nested within family ID) as random intercepts and age as random slope. Only adjusted post-hoc p values less than 0.1 are shown.

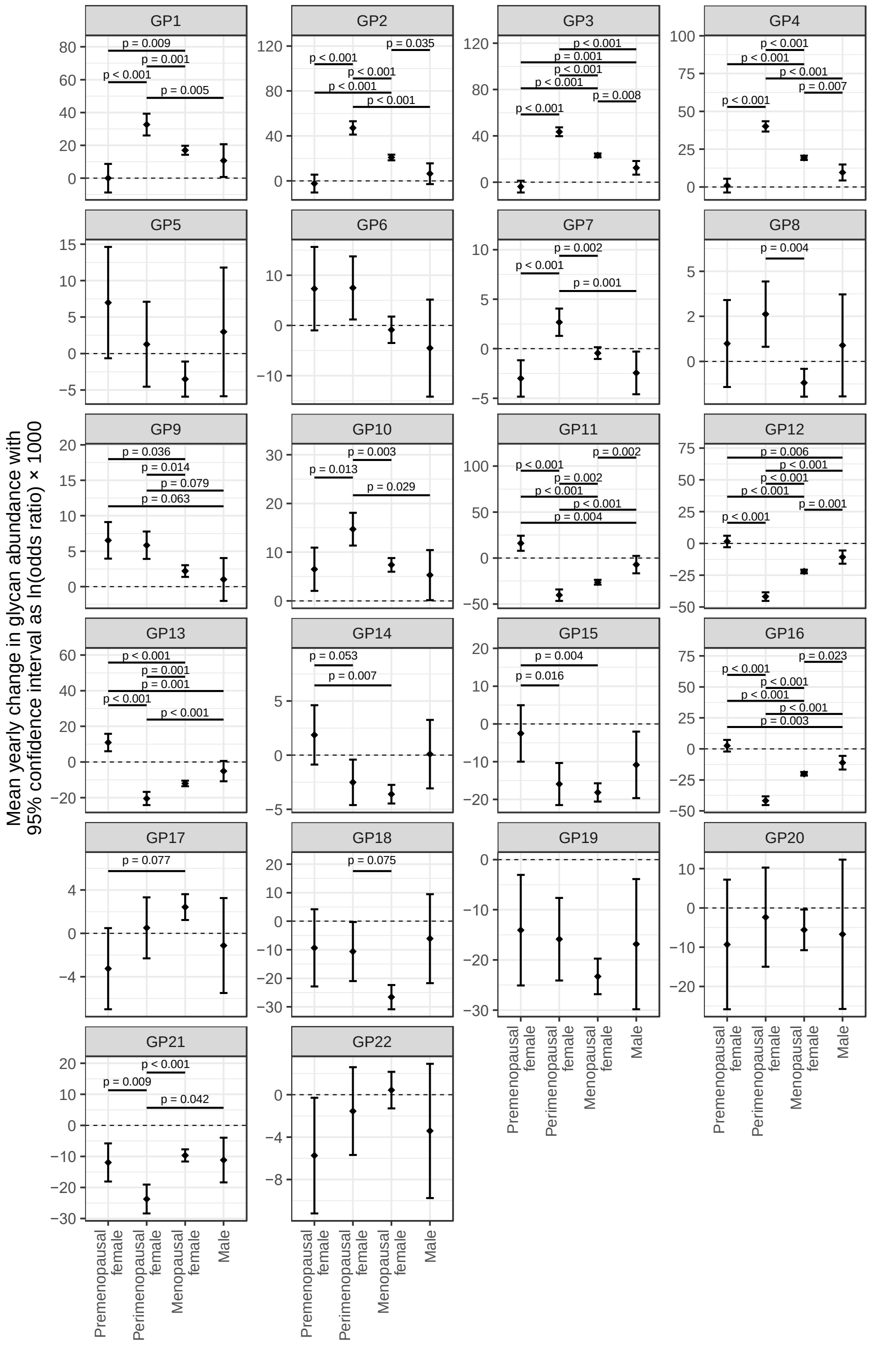

Figure S3. Average annual change of IgG glycans in females during the perimenopause period, females in pre- or postmenopause, and males. Related to Figure 2.

Mean yearly change in glycan abundances and corresponding 95% confidence intervals were estimated from the fitted mixed model with logit transformed glycan as dependent variable and sex, menopausal status (nested within sex), age and age-menopausal status interaction as fixed factors, as well as family ID and individual ID (nested within family ID) as random intercepts and age as random slope. Only adjusted post-hoc p values less than 0.1 are shown.

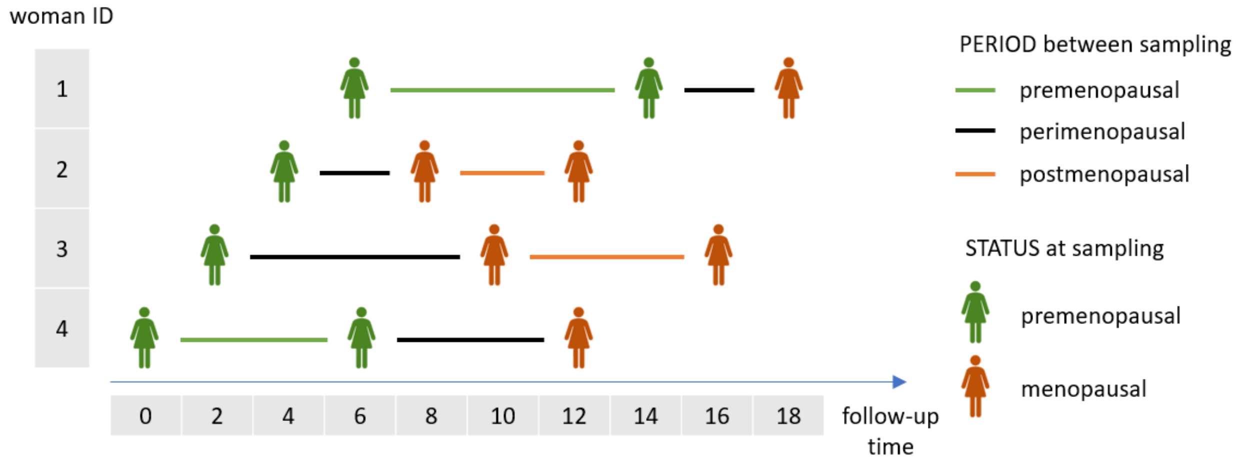

Figure S4. Diagram indicating which time points were used in calculating the yearly rate of change in IgG-N-glycome composition. Related to Figure 2.
The x-scale is expressed in years.

Table S1. Descriptive information about the cohort. Related to Figure 1 and Figure 2.

	Samples	***Individuals***	***Families***
**Count, N**	5354	2053	1173
- by sex (%)			
Male	274 (5)	113 (6)	69 (6)
Female	5080 (95)	1940 (94)	1104 (94)
- by group (%)			
Pre-menopause	1087 (21)	732	500
Menopause	3993 (79)	1678	969
**- by age subset (%)**			
Between 45 and 55 years	1374 (26)	1065 (52)	670 (57)
- by sex (%)			
Male	87 (6)	59 (6)	39 (6)
Female	1287 (94)	1006 (94)	631 (94)
- by group (%)			
Pre-menopause	507 (39)	453	340
Menopause	780 (61)	662	458
**- by number of samples (%)**			
One		203 (10)	
Two		399 (19)	
Three		1451 (71)	
**Age in years, mean (SD)**	58.3 (11.3)	57.6 (10.3)	57.2 (10.3)
- by sex			
Male	57.9 (11.7)	57.4 (10.8)	57.4 (10.9)
Female	58.3 (11.2)	57.6 (10.3)	57.2 (10.3)
- by group			
Pre-menopause	43.1 (7.1)	44.0 (6.0)	44.6 (5.9)
Menopause	62.4 (8.2)	61.7 (6.6)	61.3 (6.7)
**- by age subset**			
Between 45 and 55 years	50.6 (3.1)	50.7 (2.6)	50.6 (2.6)
- by sex			
Male	50.5 (3.1)	50.6 (2.6)	50.6 (2.5)
Female	50.6 (3.1)	50.7 (2.6)	50.6 (2.6)
- by group			
Pre-menopause	48.5 (2.6)	48.5 (2.4)	48.7 (2.4)
Menopause	51.9 (2.7)	52.2 (2.3)	52.0 (2.2)
**Follow up time in years, mean (SD)**			
**between consecutive samples**		6.94 (2.58)	
- by sex			
Male		7.17 (2.94)	
Female		6.93 (2.56)	
**total follow up time**		12.2 (4.3)	
- by sex			
Male		12.1 (4.8)	
Female		12.2 (4.3)	

Table S2. A detailed description of glycan structures. Related to Figure 1 and Figure 2. Glycan structures, corresponding to every individual immunoglobulin RapiFluor-MS labelled glycan peak, were assigned as described in Keser et al. (Keser et al., 2018).*

RapiFluor-MS glycan peak	***Glycan structure***	***Description***	***Formula***
GP1	FA1	core fucosylated, monoantennary	GP1 / GP
GP2	A2	agalactosylated, biantennary	GP2 / GP
GP3	FA2;<br>A2B	core fucosylated, biantennary (major);<br>biantennary with bisecting GlcNAc (minor)	GP3 / GP
GP4	FA2B;<br>M5	core fucosylated, biantennary with bisecting GlcNAc (major); oligomannose (minor)	GP4 / GP
GP5	A2[6]G1	monogalactosylated, biantennary	GP5 / GP
GP6	A2[3]G1	monogalactosylated, biantennary	GP6 / GP
GP7	FA2[6]G1	core fucosylated and monogalactosylated, biantennary	GP7 / GP
GP8	FA2[3]G1	core fucosylated and monogalactosylated, biantennary	GP8 / GP
GP9	FA2[6]BG1	core fucosylated and monogalactosylated, biantennary with bisecting GlcNAc	GP9 / GP
GP10	FA2[3]BG1	core fucosylated and monogalactosylated, biantennary with bisecting GlcNAc	GP10 / GP
GP11	A2G2	digalactosylated, biantennary	GP11 / GP
GP12	FA2G2;<br>A2BG2	core fucosylated, digalactosylated, biantennary (major);<br>digalactosylated, biantennary with bisecting GlcNAc (minor)	GP12 / GP
GP13	FA2BG2	core fucosylated, digalactosylated, biantennary with bisecting GlcNAc	GP13 / GP
GP14	FA2[6]G1S1;<br>FA2[3]G1S1	core fucosylated, monogalactosylated and monosialylated biantennary; core fucosylated, monogalactosylated and monosialylated biantennary	GP14 / GP
GP15	A2G2S1	digalactosylated and monosialylated biantennary	GP15 / GP
GP16	FA2G2S1	core fucosylated, digalactosylated and monosialylated biantennary	GP16 / GP
GP17	FA2BG2S1	core fucosylated, digalactosylated and monosialylated biantennary with bisecting GlcNAc	GP17 / GP
GP18	n.d.	structure not determined	GP18 / GP
GP19	A2G2S2	digalactosylated and disialylated biantennary	GP19 / GP
GP20	A2BG2S2	digalactosylated and disialylated biantennary with bisecting GlcNAc	GP20 / GP
GP21	FA2G2S2	core fucosylated, digalactosylated and disialylated biantennary	GP21 / GP
GP22	FA2BG2S2	core fucosylated, digalactosylated and disialylated biantennary with bisecting GlcNAc	GP22 / GP

*structure abbreviations – all N-glycans have two core *N*-acetylglucosamines (GlcNAcs); F at the start of the abbreviation indicates a core-fucose α1,6-linked to the inner GlcNAc; Mx, number (x) of mannose on core GlcNAcs; Ax, number of antenna (GlcNAc) on trimannosyl core; A2, biantennary with both GlcNAcs as β1,2-linked; B, bisecting GlcNAc linked β1,4 to β1,3 mannose; G(x), number (x) of β1,4 linked galactose on antenna; S(x), number (x) of sialic acids linked to galactose.

Table S3. Formulas used for the calculation of IgG derived traits. Related to Figure 1, Figure 2 and Figure 4.

Structural feature	***Formula***
Agalactosylation (G0)	GP1+GP2+GP3+GP4
Monogalactosylation (G1)	GP5+GP6+GP7+GP8+GP9
Digalactosylation (G2)	GP10+GP11+GP12+GP13
Monosialylation (S1)	GP14+GP15+GP16+GP17
Disialylation (S2)	GP19+GP20+GP21+GP22
Incidence of bisecting GlcNAc (B)	GP4+GP9+GP10+GP13+GP17+GP20+GP22
Core fucosylation (CF)	GP1+GP3+GP4+GP7+GP8+GP9+GP10+GP12+GP13+GP14+ GP16+GP17+GP21+GP22

Table S4. **Descriptive information about validation cohorts.** Related to Figure 4.

Cohort	*Premenopausal women*	*Menopausal women*
Vis		
N (%)	62 (47)	70 (53)
Age, mean (SD)	48.4 (2.9)	51.1 (3.3)
Korcula		
N (%)	86 (51)	83 (49)
Age, mean (SD)	49.5 (3.1)	52.0 (2.5)
